# What do mammals have to say about the neurobiology of acoustic communication?

**DOI:** 10.12688/molpsychol.17539.1

**Published:** 2023-05-04

**Authors:** Angeles Salles, Joshua Neunuebel

**Affiliations:** 1 Biological Sciences, University of Illinois Chicago, Chicago, Illinois, USA; 2 Psychological and Brain Sciences, University of Delaware, Newark, Delaware, USA

**Keywords:** comparative models, auditory processing, vocal production, vocalizations, social communication

## Abstract

Auditory communication is crucial across taxa, including humans, because it enables individuals to convey information about threats, food sources, mating opportunities, and other social cues necessary for survival. Comparative approaches to auditory communication will help bridge gaps across taxa and facilitate our understanding of the neural mechanisms underlying this complex task. In this work, we briefly review the field of auditory communication processing and the classical champion animal, the songbird. In addition, we discuss other mammalian species that are advancing the field. In particular, we emphasize mice and bats, highlighting the characteristics that may inform how we think about communication processing.

## Introduction

The field of neuroethology heavily relies on the natural characteristics that make different species well-suited to study particular questions. Within the field, we lean on Krogh’s principle that “*For such a large number of problems, there will be some animal of choice or a few such animals on which it can be most conveniently studied*”; now we can move a step forward and expand the champion animals for the field of auditory communication to take advantage of a comparative perspective. Other approaches focus on implementing state-of-the-art techniques on a few genetically tractable animal models, such as mice, flies, and worms. Today, we can benefit from a different mindset: new insights emerge by observing innate behaviors in classical species and applying innovative techniques, melding neuroethology and systems neuroscience. Here, we briefly review the classical animal model for the study of acoustic communication, songbirds. We will highlight the significant and fundamental groundwork this model system provided in animal communication and then advocate for how the influential discoveries serve as a springboard for studying related questions in different mammals. Broadly speaking, we will first focus on a wide range of mammals but ultimately underscore the advantages, limitations, and untapped opportunities for bats and mice.

## The backbone of the field: Songbirds

Songbirds have been the long-standing model organism for vocal learning and acoustic communication^1^. The acoustic and syntactic complexity of their songs and the ability of many avian species to learn these sounds provide exceptional correlates to human speech^2^. Song complexity has evolved under sexual selection, as most females choose mates according to song quality^3^. This evolution of song complexity produced a treasure trove of vocal repertoires exploited to study auditory signal production and processing simultaneously. For example, fundamental studies showed that maintaining a stable song depends on proper auditory feedback in several songbird species^4^. Also, many studies have described song-selective neurons in the caudomedial nidopallium and Field L, the avian homolog of the auditory cortex^5^. Neurons tuned to species-specific sounds are found in these brain regions, and the responses of these neurons depend on social context^6,7^.

These findings suggest that neural selectivity to species-specific sounds may facilitate vocal learning through auditory feedback^8^ Selectivity for conspecific calls is also evidenced by a strong response bias to natural calls instead of synthetic signals^9^. Additional studies revealed that neuronal selectivity in the caudomedial nidopallium and Field L is driven by discrete frequency components and spectral contrast of vocalizations–not by harmonicity^10^. These findings shed light on the acoustic features that may carry behavioral information. Moreover, salient acoustic features such as temporal and spectral modulations drive more robust neural responses than characteristics such as frequency modulations^11^. Though much work has focused on how acoustic features drive neuronal selectivity, it is less clear how and what specific acoustic features of natural vocalizations drive neuronal selectivity rather than synthetic stimuli. In a secondary auditory area, the medial caudal mesopallium, some neurons are tuned to specific acoustic features (motifs), and this selectivity depends on the birds’ prior experience^12^. Familiarity with vocalizations appears to be critical for selectivity in most auditory processing areas of songbird brains^13^, highlighting the importance of top-down processing in sound classification.

Birds are specialists in vocal communication and exhibit extensive repertoires of learned songs. These insights have helped start unmasking the neural mechanisms of social communication. However, limiting our focus to songbirds may constrain our understanding of the neural basis of social communication, as there are differences between avian and mammalian brains. Moreover, it is essential to bridge experimental and analytical designs to extract fundamental principles across taxa^14^.

## Here and now: Mammalian models

Acoustic communication is commonly observed across a wide range of mammals. Each species provides a robust system for elucidating various facets of how information is transferred between animals and the neural networks that underlie this sophisticated process. In humans, auditory processing of communication has focused on psychophysical studies of language perception, EEG, fMRI, and the underlying genetics of language disorders. While significant advances in our understanding of how human brains process language transpired over the last decades, the approaches have many limitations in revealing circuit mechanisms and rapid neural dynamics underlying communication processing. Thus, animal models remain a necessity in the exploration of these topics. Delphinids, a family of approximately 35 species of dolphins, have a complex, adaptable vocal repertoire and show vocal learning^15^. Moreover, the closest living terrestrial animal to these aquatic mammals is the hippopotamus^16^, thus making delphinids an ideal model for studying the evolution of vocal communication. Marmosets (*Callithrix jacchus*), New World primates, are emerging as a prominent model for studying vocal communication^17^. These primates have a rich vocal repertoire consisting of simple and compound calls^18^ that appear to be elicited or suppressed by vocal-only neurons in the premotor cortex^19^. Prairie voles (*Microtus ochrogaster*), small rodents found in North America, are a powerful model for uncovering the link between hormones, neural circuits, and social behavior. Groundbreaking work by Amadei *et al*.^20^ revealed that corticostriatal activity enhances female prairie vole huddling, an affiliative behavior believed to be regulated by oxytocin and other hormones^21,22^. Because adult prairie voles vocalize during these social interactions^23^ the possibility exists for using this mammalian model to study vocal communication in conjunction with hormones, brains, and naturalistic behavior. Naked mole rats (*Heterocephalus glaber*) are burrowing rodents native to Africa. While in physical contact with other conspecifics, these mammals utilize antiphonal calling^24^. The vocal repertoire of *Heterocephalus glaber* consists of at least 17 distinct vocalizations^25^. One category, the soft chirp, transmits information about colony membership and can be learned by pups cross-fostered in a foreign colony, thus opening the possibility of exploring whether vocal learning exists in this mammalian species^26^. Alston’s singing mice (*Scotinomys teguina*) are vocally interactive neotropical rodents. These vocal interactions are cortically dependent, temporally precise, and socially modulated^27^. Both males and females participate in vocal interactions consisting of discrete frequency-modulated harmonic broadband notes^28,29^. Because of the tight precision between calling and responding, Alston’s singing mice are an ideal model for understanding the temporal dynamics linking auditory processing and motor control.

Each of these species provides a unique advantage in studying communication processing, and a comparative approach might profoundly advance our understanding of the neural basis of social communication. In particular, two taxa of mammals in which significant progress has been made in recent years are bats and mice, and we delve into this research in the following sections.

## Mice as mammalian models for auditory communication

Like other mammalian species, mice utilize a diverse acoustic repertoire signaling with both audible, low-frequency squeaks^30^ and ultrasonic, high-frequency vocalizations^31^. Low-frequency vocalizations are typically associated with mating, pain, and fear^32,33^, but the sound’s meaning appears context-dependent. On the other hand, the purpose of mouse ultrasonic vocalizations (USVs) is an ongoing debate. The information conveyed by these signals, however, might be context-dependent. For example, when young male and female pups fall out of the nest, they emit USVs, and then dams retrieve them^34,35^. As juveniles, mice stop vocalizing, although why this vocal hiatus exists is still unclear^36^. Adult male mice produce ultrasonic vocalizations when singly housed and exposed to female urine^37^ or other scent cues^38^. Although rarer, female mice also vocalize when exposed to male urine^39^. As adult animals socialize, vocal production is prevalent when a female cohabitates with another female^40,41^. In some strains, pairing males elicits vocal production^42,43^.

During courtship, a commonly held assumption is that only male mice vocalize^44–46^. With the advent of new technology, that enables sound source separation and localization, experimenters could determine where a USV originated. This technological advancement allowed multiple groups to reveal that both sexes vocalize during courtship^47–49^. Mouse vocalizations also hold translational value. For instance, impairments in communication are characteristic of multiple neurological disorders. Groszer *et al*.^50^ showed that mice with a point mutation in *FOXP2*, a gene associated with human speech deficits^51^, emit complex innate USVs have deficits in motor skill learning, and display synaptic plasticity impairments. Chabout and colleagues^52^ extended this finding by showing that FOXP2-mutant mice produce USVs with a different temporal patterning than controls. Similarly, mouse models of autism show deviations in the acoustic features of vocalizations and emission rate compared to controls^53–55^. However, it remains an open question on how these altered patterns in vocal signaling shape behavior and what information these auditory cues convey.

Progress toward decoding the meaning of different ultrasonic vocalizations has advanced significantly after multiple groups started using molecular tools that allow precise control over specific neurons and identification of vocalizing animals. Sangiamo *et al*.^56^ demonstrated that specific ultrasonic vocal signals are associated with distant social behaviors. These signals also alter the behavior of a socially engaged partner, highlighting the communicative role mouse USVs play in social behavior. Work by Chen *et al*.^57^ and Tschida *et al*.^58^ unmasked the neural circuitry underlying USV production. Chen and colleagues showed that activating a distinct class of lateral preoptic area (LPOA) neurons elicited vocal production that resembled the repertoire of USVs produced by control animals. These LPOA neurons express oestrogen receptor 1 and project to the periaqueductal grey (PAG). Related, Tschida and colleagues demonstrated that activating PAG neurons tagged during USV emission gate affected downstream vocal-patterning circuits. These seminal discoveries depended on innovative genetic approaches to identify and control neurons in mice. While these experiments have illuminated the necessity of midbrain and hindbrain circuity in vocal production, many unresolved questions remain. For example, a long-standing theory of acoustic communication suggests that an animal’s motivational state underlies the classes of vocalizations produced^59^. Indirect evidence supports this hypothesis, but how motivation affects the circuitry controlling vocal production is unclear. By taking advantage of the tools optimized to probe the neural circuitry of mice, elucidating this mystery and many others in mammalian communication is possible.

## Bats and their contributions to understanding acoustic communication

Echolocating bats navigate in darkness by producing ultrasonic vocalizations and listening to the echoes generated by objects in their environment. Bats extract differences in echo intensity, spectrum, and binaural timing comparisons to determine the time delay between sonar emission and echo return. These timing differences allow bats to account for distance, thus accurately computing the position of prey and other objects in the environment^60–62^. Bats are audio-vocal specialists that can adapt the features of their ultrasonic vocalizations in response to the perceived environment. For example, bats change the duration and rate of echolocation calls as they approach prey^63,64^. Because bats show diverse and complex social behaviors, a vast repertoire of vocalizations (which may be learned depending on the species) and are well suited for laboratory research^65,66^, these animals have emerged as an ideal mammalian model for studying communication sound processing – not just a system for understanding echolocation. Recent work in bats has shed light on the neural mechanisms underlying the auditory processing of communication calls. In different bat species, neural selectivity for communication calls is present across brain regions; and population dynamics underlies call categorization in the auditory pathway (Inferior colliculus:^67–69^; Auditory cortex:^70–72^). This call selectivity is also present in affective processing areas such as the amygdala (Amg) and the PAG^73–76^. Current research investigates how the frontal cortex integrates auditory information to enable identity coding across individual bats interacting with conspecifics^77^. This discovery opened many questions regarding how social context modulates auditory processing.

This nascent field of auditory communication processing in bats is occurring at an ideal time, as molecular tools that had traditionally only been available for common model species (*i.e.*, mice and flies) are now available for a wider variety of species, including bats. In particular, the Bat1K project, a consortium that aims to generate chromosome-level genomes for all bat species, sequenced the genomes from 21 species^78^. This effort has enabled the first transgenic bat, allowing researchers to manipulate the expression of FoxP2^79^. In addition to target genes like FOXP2, other key molecules involved in the modulation of communication call signal processing are hormones and neurotransmitters. Dopamine, norepinephrine, serotonin, corticosterone, and adrenocorticotropic hormone release in the Amg increases in *Cynopterus brachyotis* bats (a bat species that does not use echolocation) when they produce or hear multi-harmonic distress calls^80^. Further, the distress calls in *Cynopterus brachyotis* elevate the levels of different proteins (TH, Nurr-1, DAT, D1DR) in the Amg of both the emitter and receiver engaged in live interactions, but not in bats listening passively to playback of modified distress calls^81^. These studies open the field to molecular approaches in bats that can lead the way for future studies across species.

Lastly, some bat species are vocal learners, a rare trait in the animal kingdom and valuable for studying the neural mechanisms of auditory communication. Young *Saccopteryx bilineata* bats learn their communication calls and practice their vocalizations similar to human infants during the babbling phase^82,83^. *Rousettus aegyptiacus* learn their vocalizations from their colony mates and can modify the acoustic parameters of their calls^84,85^. *Phyllostomus discolor* bats also learn their social vocalizations^86^, and modify acoustic parameters based on playbacks of communication calls^87^.

These studies pave the way for solidifying bats as mammalian models to study the neural mechanisms of auditory communication. Their expansive vocalization repertoires, complex social behaviors, similarities in brain structures with other mammals, and adaptability to laboratory life make bats strong candidates in the pursuit of comparative models in the field of auditory communication.

## Looking to the future of comparative approaches

**Figure 1.**
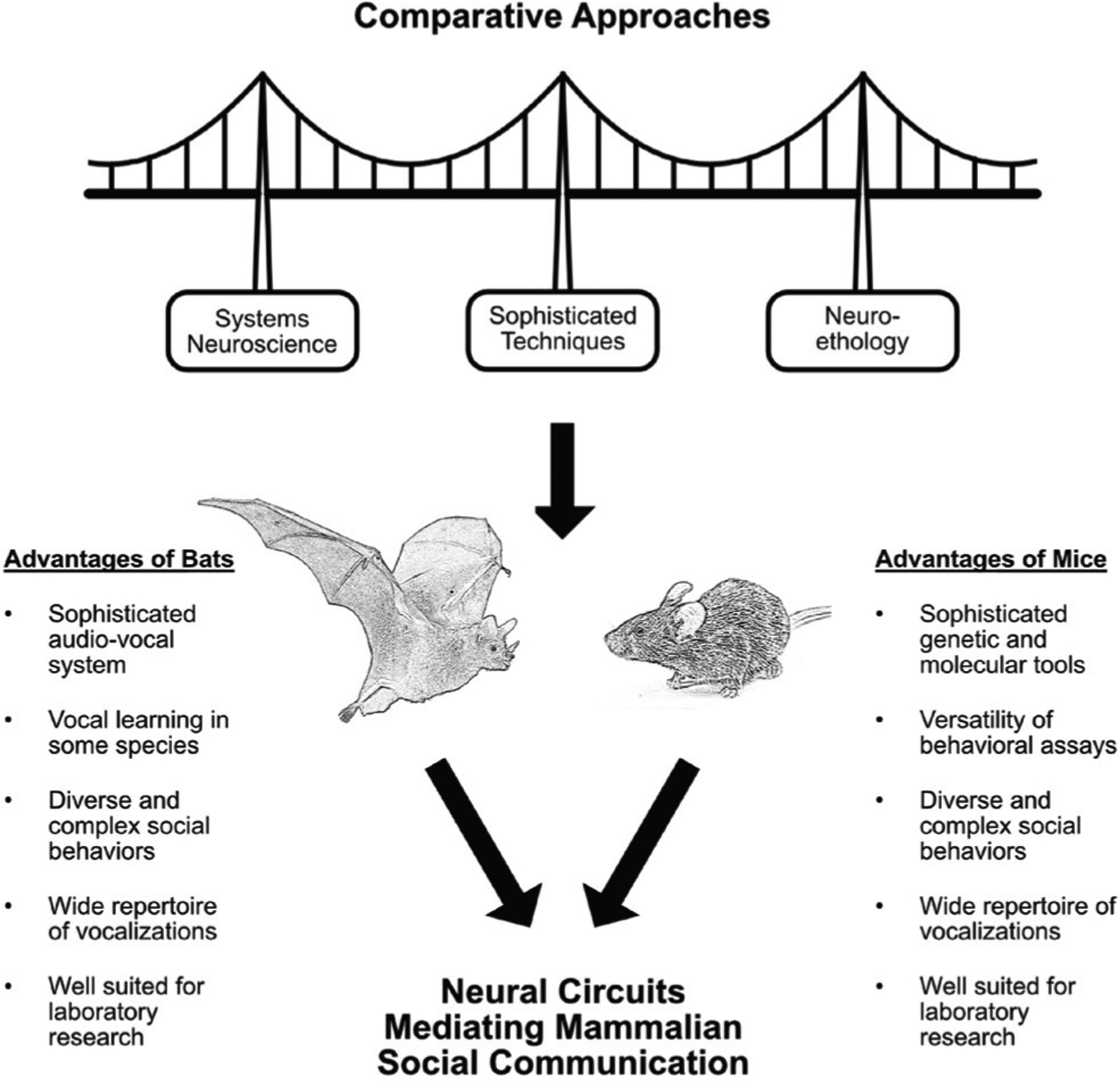
Graphical abstract of the proposed advantages of comparative work in the field of neural circuits mediating mammalian social communication. We define sophisticated techniques as chemogenetics, intersectional genetics, single-cell resolution connectomics, spatial transcriptomics, and computational approaches that will help bridge system neuroscience and neuroethology.

The field of auditory communication has seen significant advances fueled by the birdsong system. Nowadays, the use of mammals to study social communication is expanding, and collaborations among researchers using different models can help answer fundamental questions about the parity of systems across taxonomic groups. Specifically, employing the same approaches for a one-to-one comparison can provide insights and open new avenues for the field (Figure 1).

**Figure 2.**
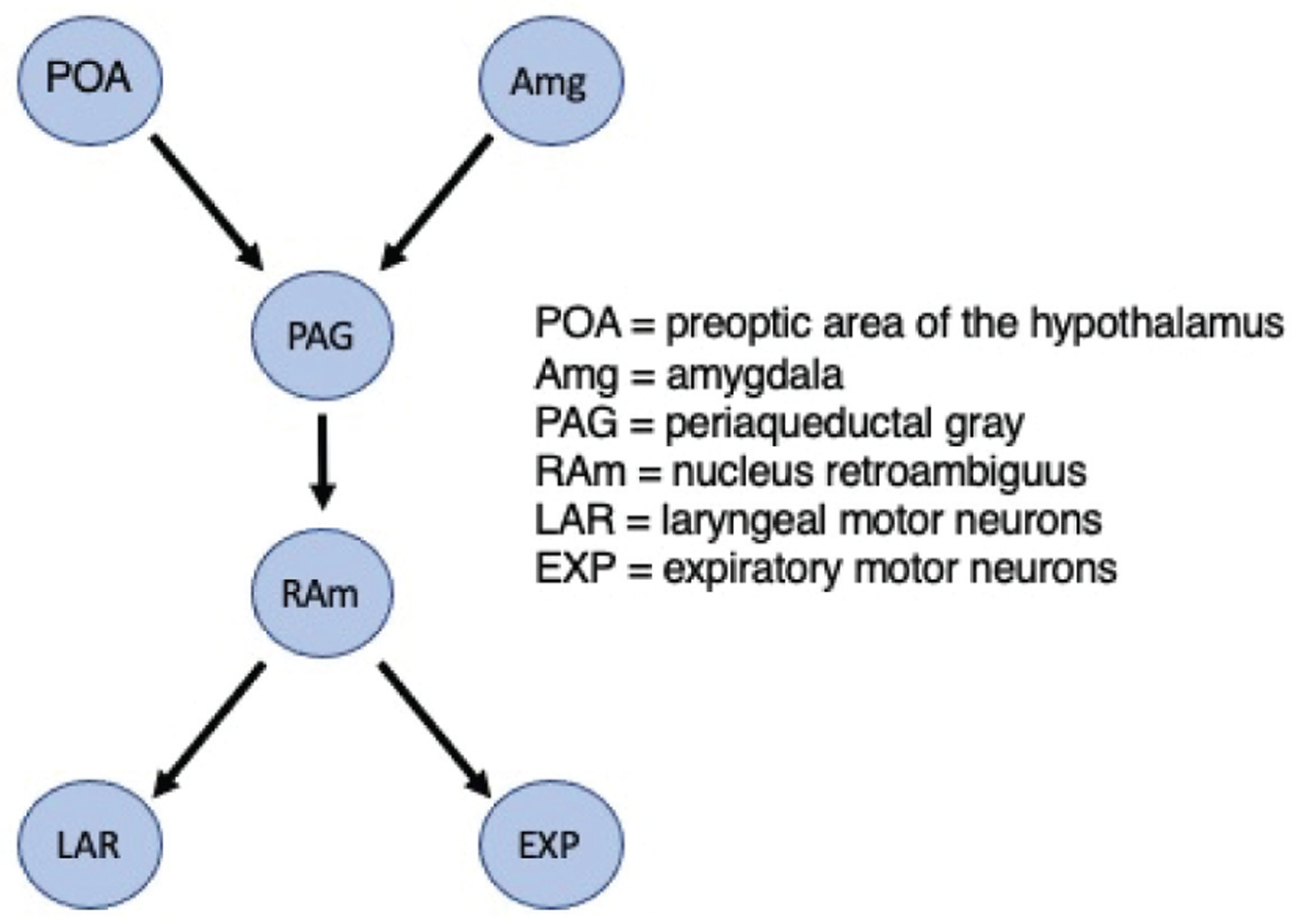
Diagram showing proposed circuitry underlying vocal production in mammals.

One potential example utilizing a comparative approach is to study the circuitry involved in auditory communication across species. Specifically, we emphasize the need and benefits of comparing the conserved neural networks underlying vocal production and audio-vocal integration, focusing on the PAG. In mammals, the PAG receives input from the POA and the Amg and projects to laryngeal and expiratory motor neurons^88^; thus, this neural structure is ideally positioned to play a crucial role in modulating the vocal emission of social calls (Figure 2). Microstimulation experiments show that the PAG is involved in pathways that control the production of bat communication vocalizations^76^. Valentine *et al*.^89^ extended this finding and showed that distinct regions of the PAG appear to be dedicated to producing bat communication sounds.

Direct simulations of specific PAG neurons elicited USV production in male and female mice^58^. Michael *et al*.^90^ found that activating PAG-projecting neurons in the POA and central-medial boundary zone of the Amg stimulated and suppressed mouse USV production, respectively. In both species, direct evidence exists supporting that PAG is essential for the emission of social calls. One could then leverage the fact that bats also emit echolocation calls while mice do not echolocate. This difference opens the possibility of examining parallel circuitry that might underlie different modes of vocal emission and answer questions that could elucidate separate neural architectures for echolocation. The potential is intriguing, as some research supports the idea that parallel pathways are in place to produce echolocation and communication calls in bats. For instance, stimulation of the paralemniscal area (PLA), situated around the nuclei of the lateral lemniscus in the ventral midbrain, only elicits echolocation calls^76^. However, the production of communication calls through microstimulation of the PAG is not affected by PLA inhibition^91^. Like bats, mice also have a PLA, but the functional relevance to vocal emission is unknown. These similarities and differences in vocal emission and neural circuitry beg for further investigation into the connectivity, neuronal type, and circuit layout in bats compared to other animals allowing us to explore the potential conserved systems in producing and processing communication calls.

## Concluding remarks

The advent of technologies enabling the exploration of questions in neuroscience across taxa has exciting implications for the field. Molecular, cellular, and behavioral techniques provide the toolkit to dissect the mechanisms by which animals produce and process communication. Bats, mice, and other mammalian models can build on the extensive research done by the bird song community, thus improving our understanding of the neural architecture underlying this complex system. Similarities across species will enable us to generalize and seek specific targets for research in other models, such as humans. For example, studying genes identified as potential targets in humans with communication disorders and dissecting their role in acoustic communication in parallel across mammalian species may reveal therapeutic candidates to improve patients’ daily lives. Also, identifying the differences across taxa in how diverse species process communication sounds can provide insights into convergent evolutionary traits that are supported by different neural scaffolding. Furthermore, from an environmental perspective, knowing more about how different animals communicate and carry out their social interactions will provide vital information for future conservation efforts. Lastly, merging the fields of systems neuroscience and neuroethology promises a mutually beneficial interaction where auditory communication can be explored in diverse animal models especially suited for the questions at hand, while employing state-of-the-art and well-established techniques.

## Data Availability

No data are associated with this article.

## Amendments from Version 1

In this revised version of the manuscript, we addressed the reviewers comments as detailed in the response to reviewer 1. Briefly, we have changed the word species for strains when speaking of different mus musculus lab mice. We also clarified that the technological advances we refer to are those that enable sound source separation and localization. We’ve also reworded the sentence referring to the work on a point mutation FOXP2 in mice to more accurately describe the work of Groszer *et al*. We’ve clarified the location of the parameniscal (PLA) area and elaborated the figure legend of Figure 1 to enumerate the sophisticated techniques that are advancing comparative studies.

Any further responses from the reviewers can be found at the end of the article

## References

[1] Saito N, Maekawa M: Birdsong: the interface with human language. Brain Dev. 1993; 15(1): 31–39. 8338209 10.1016/0387-7604(93)90004-r

[2] Aamodt CM, Farias-Virgens M, White SA: Birdsong as a window into language origins and evolutionary neuroscience. Philos Trans R Soc Lond B Biol Sci. 2020; 375(1789): 20190060. 31735151 10.1098/rstb.2019.0060 PMC6895547

[3] Nowicki S, Searcy WA, Hughes M, : The evolution of bird song: male and female response to song innovation in swamp sparrows. Anim Behav. 2001; 62(6): 1189–1195.

[4] Brainard MS, Doupe AJ: Interruption of a basal ganglia-forebrain circuit prevents plasticity of learned vocalizations. Nature. 2000; 404(6779): 762–766. 10783889 10.1038/35008083

[5] Prather JF: Auditory signal processing in communication: perception and performance of vocal sounds. Hear Res. 2013; 305: 144–155. 23827717 10.1016/j.heares.2013.06.007 PMC3818290

[6] Pinaud R, Terleph TA: A songbird forebrain area potentially involved in auditory discrimination and memory formation. J Biosci. 2008; 33(1): 145–155. 18376079 10.1007/s12038-008-0030-y

[7] Vignal C, Andru J, Mathevon N: Social context modulates behavioural and brain immediate early gene responses to sound in male songbird. Eur J Neurosci. 2005; 22: 949–955. 16115218 10.1111/j.1460-9568.2005.04254.x

[8] Prather JF, Mooney R: Neural correlates of learned song in the avian forebrain: simultaneous representation of self and others. Curr Opin Neurobiol. 2004; 14(4): 496–502. 15321071 10.1016/j.conb.2004.06.004

[9] Grace JA, Amin N, Singh NC, : Selectivity for Conspecific Song in the Zebra Finch Auditory Forebrain. J Neurophysiol. 2003; 89(1): 472–487. 12522195 10.1152/jn.00088.2002

[10] So NLT, Edwards JA, Woolley SMN: Auditory Selectivity for Spectral Contrast in Cortical Neurons and Behavior. J Neurosci. 2020; 40(5): 1015–1027. 31826944 10.1523/JNEUROSCI.1200-19.2019 PMC6989003

[11] Singh NC, Theunissen FE: Modulation spectra of natural sounds and ethological theories of auditory processing. J Acoust Soc Am. 2003; 114(6 Pt 1): 3394–3411. 14714819 10.1121/1.1624067

[12] Gentner TQ, Hulse SH: Female European starling preference and choice for variation in conspecific male song. Anim Behav. 2000; 59(2): 443–458. 10675267 10.1006/anbe.1999.1313

[13] Janata P, Margoliash D: Gradual Emergence of Song Selectivity in Sensorimotor Structures of the Male Zebra Finch Song System. J Neurosci. 1999; 19(12): 5108–5118. 10366643 10.1523/JNEUROSCI.19-12-05108.1999 PMC6782639

[14] Woolley SMN, Portfors CV: Conserved mechanisms of vocalization coding in mammalian and songbird auditory midbrain. Hear Res. 2013; 305: 45–56. 23726970 10.1016/j.heares.2013.05.005 PMC3818289

[15] Janik VM: Chapter 4 Acoustic Communication in Delphinids. Adv Study Behav. Academic Press, 2009; 40: 123–157.

[16] Ursing BM, Arnason U: Analyses of mitochondrial genomes strongly support a hippopotamus-whale clade. Proc Biol Sci. 1998; 265(1412): 2251–2255. 9881471 10.1098/rspb.1998.0567 PMC1689531

[17] Eliades SJ, Miller CT: Marmoset vocal communication: Behavior and neurobiology. Dev Neurobiol. 2017; 77(3): 286–299. 27739195 10.1002/dneu.22464

[18] Agamaite JA, Chang CJ, Osmanski MS, : A quantitative acoustic analysis of the vocal repertoire of the common marmoset (*Callithrix jacchus*). J Acoust Soc Am. 2015; 138(5): 2906–2928. 26627765 10.1121/1.4934268 PMC4644241

[19] Roy S, Zhao L, Wang X: Distinct Neural Activities in Premotor Cortex during Natural Vocal Behaviors in a New World Primate, the Common Marmoset (*Callithrix jacchus*). J Neurosci. 2016; 36(48): 12168–12179. 27903726 10.1523/JNEUROSCI.1646-16.2016 PMC5148218

[20] Amadei EA, Johnson ZV, Kwon YJ, : Dynamic corticostriatal activity biases social bonding in monogamous female prairie voles. Nature. 2017; 546(7657): 297–301. 28562592 10.1038/nature22381 PMC5499998

[21] Witt DM, Carter CS, Walton DM: Central and peripheral effects of oxytocin administration in prairie voles (*Microtus ochrogaster*). Pharmacol Biochem Behav. 1990; 37(1): 63–69. 2263668 10.1016/0091-3057(90)90042-g

[22] Berendzen KM, Sharma R, Mandujano MA, : Oxytocin receptor is not required for social attachment in prairie voles. Neuron. 2023; 111(6): 787–796.e4. 36708707 10.1016/j.neuron.2022.12.011 PMC10150797

[23] Lepri JJ, Theodorides M, Wysocki CJ: Ultrasonic vocalizations by adult prairie voles, *Microtus ochrogaster*. Experientia. 1988; 44(3): 271–273. 3280341 10.1007/BF01941736

[24] Yosida S, Kobayasi KI, Ikebuchi M, : Antiphonal Vocalization of a Subterranean Rodent, the Naked Mole-Rat (*Heterocephalus glaber*). Ethology. 2007; 113(7): 703–710.

[25] Sherman PW, Jarvis JUM, Alexander RD: The Biology of the Naked Mole-Rat. Princeton University Press, 2017.

[26] Barker AJ, Veviurko G, Bennett NC, : Cultural transmission of vocal dialect in the naked mole-rat. Science. 2021; 371(6528): 503–507. 33510025 10.1126/science.abc6588

[27] Okobi DE Jr, Banerjee A, Matheson AMM, : Motor cortical control of vocal interaction in neotropical singing mice. Science. 2019; 363(6430): 983–988. 30819963 10.1126/science.aau9480

[28] Campbell P, Pasch B, Pino JL, : Geographic Variation in the Songs of Neotropical Singing Mice: Testing the Relative Importance of Drift and Local Adaptation. Evolution. 2010; 64(7): 1955–1972. 20148958 10.1111/j.1558-5646.2010.00962.x

[29] Miller JR, Engstrom MD: Vocal Stereotypy and Singing Behavior in Baiomyine Mice. J Mammal. 2007; 88(6): 1447–1465.

[30] Grimsley JMS, Hazlett EG, Wenstrup JJ: Coding the meaning of sounds: contextual modulation of auditory responses in the basolateral amygdala. J Neurosci. 2013; 33(44): 17538–17548. 24174686 10.1523/JNEUROSCI.2205-13.2013 PMC3812514

[31] Sales GD (Neé Sewell): Ultrasound and mating behaviour in rodents with some observations on other behavioural situations. J Zool. 1972; 168(2): 149–164.

[32] Wang H, Liang S, Burgdorf J, : Ultrasonic vocalizations induced by sex and amphetamine in M2, M4, M5 muscarinic and D2 dopamine receptor knockout mice. PLoS One. 2008; 3(4): e1893. 18382674 10.1371/journal.pone.0001893 PMC2268741

[33] Williams WO, Riskin DK, Mott AKM: Ultrasonic sound as an indicator of acute pain in laboratory mice. J Am Assoc Lab Anim Sci. 2008; 47(1): 8–10. PMC2652617 18210991

[34] Shepard KN, Lin FG, Zhao CL, : Behavioral Relevance Helps Untangle Natural Vocal Categories in a Specific Subset of Core Auditory Cortical Pyramidal Neurons. J Neurosci. 2015; 35(6): 2636–2645. 25673855 10.1523/JNEUROSCI.3803-14.2015 PMC4323536

[35] Marlin BJ, Mitre M, D’amour JA, : Oxytocin enables maternal behaviour by balancing cortical inhibition. Nature. 2015; 520(7548): 499–504. 25874674 10.1038/nature14402 PMC4409554

[36] Grimsley JMS, Monaghan JJM, Wenstrup JJ, : Development of social vocalizations in mice. PLoS One. 2011; 6(3): e17460. 21408007 10.1371/journal.pone.0017460 PMC3052362

[37] Holy TE, Guo Z: Ultrasonic Songs of Male Mice. PLoS Biol. 2005; 3(12): e386. 16248680 10.1371/journal.pbio.0030386 PMC1275525

[38] Whitney G, Alpern M, Dizinno G, : Female odors evoke ultrasounds from male mice. Anim Learn Behav. 1974; 2(1): 13–18. 4468889 10.3758/bf03199109

[39] Ronald KL, Zhang X, Morrison MV, : Male mice adjust courtship behavior in response to female multimodal signals. PLoS One. 2020; 15(4): e0229302. 32241020 10.1371/journal.pone.0229302 PMC7117945

[40] Zala SM, Reitschmidt D, Noll A, : Sex-dependent modulation of ultrasonic vocalizations in house mice (*Mus musculus musculus*). PLoS One. 2017; 12(12): e0188647. 29236704 10.1371/journal.pone.0188647 PMC5728457

[41] Maggio JC, Whitney G: Ultrasonic vocalizing by adult female mice (Mus musculus). J Comp Psychol. 1985; 99(4): 420–436. 4075780

[42] Seagraves KM, Arthur BJ, Roian Egnor SE, : Evidence for an audience effect in mice: male social partners alter the male vocal response to female cues. J Exp Biol. 2016; 219(Pt 10): 1437–1448. 27207951 10.1242/jeb.129361 PMC4874560

[43] Keesom SM, Finton CJ, Sell GL, : Early-Life Social Isolation Influences Mouse Ultrasonic Vocalizations during Male-Male Social Encounters. PLoS One. 2017; 12(1): e0169705. 28056078 10.1371/journal.pone.0169705 PMC5215938

[44] Whitney G, Coble JR, Stockton MD, : Ultrasonic emissions: do they facilitate courtship of mice. J Comp Physiol Psychol. 1973; 84(3): 445–452. 4745813 10.1037/h0034899

[45] Warburton VL, Sales GD, Milligan SR, : The emission and elicitation of mouse ultrasonic vocalizations: the effects of age, sex and gonadal status. Physiol Behav. 1989; 45(1): 41–47. 2727141 10.1016/0031-9384(89)90164-9

[46] Barthelemy M, Gourbal BEF, Gabrion C, : Influence of the female sexual cycle on BALB/c mouse calling behaviour during mating. Naturwissenschaften. 2004; 91(3): 135–138. 15034664 10.1007/s00114-004-0501-4

[47] Neunuebel JP, Taylor AL, Arthur BJ, : Female mice ultrasonically interact with males during courtship displays. Elife. 2015; 4: e06203. 26020291 10.7554/eLife.06203 PMC4447045

[48] Heckman J, McGuinness B, Celikel T, : Determinants of the mouse ultrasonic vocal structure and repertoire. Neurosci Biobehav Rev. 2016; 65: 313–325. 27060755 10.1016/j.neubiorev.2016.03.029

[49] Warren MR, Sangiamo DT, Neunuebel JP, : High Channel Count Microphone Array Accurately and Precisely Localizes Ultrasonic Signals from Freely-Moving Mice. J Neurosci Methods. 2018; 297: 44–60. 29309793 10.1016/j.jneumeth.2017.12.013 PMC8256447

[50] Groszer M, Keays DA, Deacon RMJ, : Impaired synaptic plasticity and motor learning in mice with a point mutation implicated in human speech deficits. Curr Biol. 2008; 18(5): 354–362. 18328704 10.1016/j.cub.2008.01.060 PMC2917768

[51] Lai CS, Fisher SE, Hurst JA, : A forkhead-domain gene is mutated in a severe speech and language disorder. Nature. 2001; 413(6855): 519–523. 11586359 10.1038/35097076

[52] Chabout J, Sarkar A, Patel SR, : A Foxp2 Mutation Implicated in Human Speech Deficits Alters Sequencing of Ultrasonic Vocalizations in Adult Male Mice. Front Behav Neurosci. 2016; 10: 197. 27812326 10.3389/fnbeh.2016.00197 PMC5071336

[53] Ey E, Torquet N, Le Sourd AM, : The Autism *ProSAP1/Shank2* mouse model displays quantitative and structural abnormalities in ultrasonic vocalisations. Behav Brain Res. 2013; 256: 677–689. 23994547 10.1016/j.bbr.2013.08.031

[54] Hodges SL, Nolan SO, Reynolds CD, : Spectral and temporal properties of calls reveal deficits in ultrasonic vocalizations of adult Fmr1 knockout mice. Behav Brain Res. 2017; 332: 50–58. 28552599 10.1016/j.bbr.2017.05.052 PMC6503674

[55] Wöhr M, Roullet FI, Crawley JN, : Reduced scent marking and ultrasonic vocalizations in the BTBR T+tf/J mouse model of autism. Genes Brain Behav. 2011; 10(1): 35–43. 20345893 10.1111/j.1601-183X.2010.00582.x PMC2903641

[56] Sangiamo DT, Warren MR, Neunuebel JP, : Ultrasonic signals associated with different types of social behavior of mice. Nat Neurosci. 2020; 23(3): 411–422. 32066980 10.1038/s41593-020-0584-z PMC7065962

[57] Chen J, Markowitz JE, Lilascharoen V, : Flexible scaling and persistence of social vocal communication. Nature. 2021; 593(7857): 108–113. 33790464 10.1038/s41586-021-03403-8 PMC9153763

[58] Tschida K, Michael V, Takatoh J, : A Specialized Neural Circuit Gates Social Vocalizations in the Mouse. Neuron. 2019; 103(3): 459–472.e4. 31204083 10.1016/j.neuron.2019.05.025 PMC6687542

[59] Morton ES: On the Occurrence and Significance of Motivation-Structural Rules in Some Bird and Mammal Sounds. Am Nat. 1977; 111(981): 855–869.

[60] Simmons JA: The resolution of target range by echolocating bats. J Acoust Soc Am. 1973; 54(1): 157–173. 4738624 10.1121/1.1913559

[61] Grinnell AD: The neurophysiology of audition in bats: resistance to interference. J Physiol. 1963; 167(1): 114–27. 13950556 10.1113/jphysiol.1963.sp007135 PMC1359487

[62] Aytekin M, Grassi E, Sahota M, : The bat head-related transfer function reveals binaural cues for sound localization in azimuth and elevation. J Acoust Soc Am. 2004; 116(6): 3594–605. 15658710 10.1121/1.1811412

[63] Schnitzler HU, Kalko EKV: Echolocation by Insect-Eating Bats: We define four distinct functional groups of bats and find differences in signal structure that correlate with the typical echolocation tasks faced by each group. BioScience. 2001; 51(7): 557–569.

[64] Moss CF, Surlykke A: Auditory scene analysis by echolocation in bats. J Acoust Soc Am. 2001; 110(4): 2207–2226. 11681397 10.1121/1.1398051

[65] Salles A, Bohn KM, Moss CF: Auditory communication processing in bats: What we know and where to go. Behav Neurosci. 2019; 133(3): 305–319. 31045392 10.1037/bne0000308

[66] Montoya J, Lee Y, Salles A: Social Communication in Big Brown Bats. Front Ecol Evol. 2022; 10. 10.3389/fevo.2022.903107 PMC10720915 38098690

[67] Andoni S, Pollak GD: Selectivity for spectral motion as a neural computation for encoding natural communication signals in bat inferior colliculus. J Neurosci. 2011; 31(46): 16529–40. 22090479 10.1523/JNEUROSCI.1306-11.2011 PMC3271015

[68] Portfors CV: Combination sensitivity and processing of communication calls in the inferior colliculus of the Moustached Bat Pteronotus parnellii. An Acad Bras Cienc. 2004; 76(2): 253–7. 15258635 10.1590/s0001-37652004000200010

[69] Salles A, Park S, Sundar H, : Neural Response Selectivity to Natural Sounds in the Bat Midbrain. Neuroscience. 2020; 434: 200–211. 31918008 10.1016/j.neuroscience.2019.11.047

[70] Martin LM, García-Rosales F, Beetz MJ, : Processing of temporally patterned sounds in the auditory cortex of Seba’s short-tailed bat, *Carollia perspicillata*. Eur J Neurosci. 2017; 46(8): 2365–2379. 28921742 10.1111/ejn.13702

[71] Washington SD, Kanwal JS: DSCF neurons within the primary auditory cortex of the mustached bat process frequency modulations present within social calls. J Neurophysiol. 2008; 100(6): 3285–3304. 18768643 10.1152/jn.90442.2008 PMC2604848

[72] García-Rosales F, Beetz MJ, Cabral-Calderin Y, : Neuronal coding of multiscale temporal features in communication sequences within the bat auditory cortex. Commun Biol. 2018; 1: 200. 30480101 10.1038/s42003-018-0205-5 PMC6244232

[73] Gadziola MA, Grimsley JMS, Shanbhag SJ, : A novel coding mechanism for social vocalizations in the lateral amygdala. J Neurophysiol. 2012; 107(4): 1047–1057. 22090463 10.1152/jn.00422.2011 PMC3289453

[74] Gadziola MA, Shanbhag SJ, Wenstrup JJ: Two distinct representations of social vocalizations in the basolateral amygdala. J Neurophysiol. 2016; 115(2): 868–886. 26538612 10.1152/jn.00953.2015 PMC4839489

[75] Naumann RT, Kanwal JS: Basolateral amygdala responds robustly to social calls: spiking characteristics of single unit activity. J Neurophysiol. 2011; 105(5): 2389–2404. 21368003 10.1152/jn.00580.2010 PMC3094175

[76] Fenzl T, Schuller G: Periaqueductal gray and the region of the paralemniscal area have different functions in the control of vocalization in the neotropical bat, *Phyllostomus discolor*. Eur J Neurosci. 2002; 16(10): 1974–1986. 12453061 10.1046/j.1460-9568.2002.02261.x

[77] Rose MC, Styr B, Schmid TA, : Cortical representation of group social communication in bats. Science. 2021; 374(6566): eaba9584. 34672724 10.1126/science.aba9584 PMC8775406

[78] Teeling EC, Vernes SC, Dávalos LM, : Bat Biology, Genomes, and the Bat1K Project: To Generate Chromosome-Level Genomes for All Living Bat Species. Annu Rev Anim Biosci. 2018; 6: 23–46. 29166127 10.1146/annurev-animal-022516-022811

[79] Vernes SC, Devanna P, Hörpel SG, : The pale spear-nosed bat: A neuromolecular and transgenic model for vocal learning. Ann N Y Acad Sci. 2022; 1517(1): 125–142. 36069117 10.1111/nyas.14884 PMC9826251

[80] Mariappan S, Bogdanowicz W, Marimuthu G, : Distress calls of the greater short-nosed fruit bat Cynopterus sphinx activate hypothalamic-pituitary-adrenal (HPA) axis in conspecifics. J Comp Physiol A Neuroethol Sens Neural Behav Physiol. 2013; 199(9): 775–783. 23832467 10.1007/s00359-013-0838-2

[81] Mariappan S, Bogdanowicz W, Raghuram H, : Structure of distress call: implication for specificity and activation of dopaminergic system. J Comp Physiol A Neuroethol Sens Neural Behav Physiol. 2016; 202(1): 55–65. 26610332 10.1007/s00359-015-1053-0

[82] Knörnschild M, Nagy M, Metz M, : Complex vocal imitation during ontogeny in a bat. Biol Lett. 2010; 6(2): 156–9. 19812069 10.1098/rsbl.2009.0685 PMC2865031

[83] Fernandez AA, Burchardt LS, Nagy M, : Babbling in a vocal learning bat resembles human infant babbling. Science. 2021; 373(6557): 923–926. 34413237 10.1126/science.abf9279

[84] Prat Y, Taub M, Yovel Y: Vocal learning in a social mammal: Demonstrated by isolation and playback experiments in bats. Sci Adv. 2015; 1(2): e1500019. 26601149 10.1126/sciadv.1500019 PMC4643821

[85] Prat Y, Taub M, Pratt E, : An annotated dataset of Egyptian fruit bat vocalizations across varying contexts and during vocal ontogeny. Sci Data. 2017; 4: 170143. 28972574 10.1038/sdata.2017.143 PMC5625625

[86] Esser KH: Audio-vocal learning in a non-human mammal: the lesser spear-nosed bat *Phyllostomus discolor*. Neuroreport. 1994; 5(14): 1718–20. 7827315 10.1097/00001756-199409080-00007

[87] Lattenkamp EZ, Vernes SC, Wiegrebe L: Volitional control of social vocalisations and vocal usage learning in bats. J Exp Biol. 2018; 221(Pt 14): jeb180729. 29880634 10.1242/jeb.180729

[88] Jürgens U: Neuronal Control of Mammalian Vocalization, with Special Reference to the Squirrel Monkey. Naturwissenschaften. 1998; 85(8): 376–88. 9762689 10.1007/s001140050519

[89] Valentine DE, Sinha SR, Moss CF: Orienting responses and vocalizations produced by microstimulation in the superior colliculus of the echolocating bat, *Eptesicus fuscus*. J Comp Physiol A Neuroethol Sens Neural Behav Physiol. 2002; 188(2): 89–108. 11919691 10.1007/s00359-001-0275-5

[90] Michael V, Goffinet J, Pearson J, : Circuit and synaptic organization of forebrain-to-midbrain pathways that promote and suppress vocalization. eLife. 2020; 9: e63493. 33372655 10.7554/eLife.63493 PMC7793624

[91] Fenzl T, Schuller G: Echolocation calls and communication calls are controlled differentially in the brainstem of the bat *Phyllostomus discolor*. BMC Biol. 2005; 3: 17. 16053533 10.1186/1741-7007-3-17 PMC1190161

